# Diet as an Optional Treatment in Adults With Inflammatory Bowel Disease: A Systematic Review of the Literature

**DOI:** 10.7759/cureus.42057

**Published:** 2023-07-18

**Authors:** Arturo P Jaramillo, Abdelrahman Abaza, Faten Sid Idris, Humna Anis, Ilma Vahora, Kiran Prasad Moparthi, Majdah T Al Rushaidi, MeghanaReddy Muddam, Omobolanle A Obajeun

**Affiliations:** 1 General Practice, California Institute of Behavioral Neurosciences and Psychology, Fairfield, USA; 2 Pathology, California Institute of Behavioral Neurosciences and Psychology, Fairfield, USA; 3 Pediatrics, California Institute of Behavioral Neurosciences and Psychology, Fairfield, USA; 4 Pediatrics, Combined Military Hospital (CMH) Lahore Medical College and Institute of Dentistry, Lahore, PAK; 5 General Surgery, Saint George's University School of Medicine, Chicago, USA; 6 Medicine, Sri Venkata Sai (SVS) Medical College, Mahabubnagar, Mahabubnagar, IND; 7 Geriatrics, Hamad Medical Corporation, Doha, QAT; 8 Medicine, Sri Venkata Sai (SVS) Medical College, Hyderabad, Hyderabad, IND; 9 Paediatrics, Al Zahra Private Hospital, Dubai, ARE

**Keywords:** ulcerative colıtıs, chron’s disease, (ibd) inflammatory bowel disease, ibd management, exclusive enteral nutrition, fodmap diet

## Abstract

While the exact cause of IBD is unknown, there are a number of factors that are thought to contribute to its development, including environmental and genetic factors. While exclusive enteral nutrition (EEN) is a promising therapy for Crohn's disease (CD), it is not yet considered a first-line treatment. Additionally, the efficacy of EEN compared to corticosteroid treatment is still being investigated. EEN is suggested as a first-line therapy by which guidelines and in which age groups, as it may differ in pediatric and adult recommendations. Another finding was that dietary changes involving an increase in anti-inflammatory foods and decreased intake of foods high in inflammatory compounds are linked to a beneficial outcome both metabolically and microbiologically in patients with ulcerative colitis (UC) in remission.

For relevant medical literature, we examined PubMed/Medline, the Cochrane Library, and Google Scholar as examples of medical databases. The articles were identified, evaluated, and eligibility applied, and nine publications were found. The finished articles investigated the role of several diet alternatives for patients with IBD. Some others have shown that following a normal low-fat diet may be effective in reducing the occurrence of subclinical colitis. The EEN and partial enteral nutrition (PEN) indicated no significant differences between both regimens, but both had good outcomes during active IBD. Other strict diets, such as the specific carbohydrate diet (SCD) versus the Mediterranean diet (MD), demonstrate excellent outcomes in patients with IBD. Fermentable oligosaccharides, disaccharides, monosaccharides, and polyols (FODMAP) dietary counseling improves gastrointestinal symptoms and quality of life in IBD patients. Based on the above, we concluded that more studies determining which component of the diet is not clear (proteins, carbs balanced) or diet types are required to establish a particular diet employed as a treatment intervention in these individuals.

## Introduction and background

Crohn's disease (CD) and ulcerative colitis (UC) are linked with significant morbidity and higher healthcare costs. The current model of CD pathogenesis implies that environmental variables and the gut microbiota interact in those who are genetically predisposed to the condition [1]. Steroids have historically served as the cornerstone of CD therapy. However, while steroids are effective, long-term use develops significant adverse effects compared with anti-tumor necrosis factor (TNF) therapy [2]. Tofacitinib, a Janus kinase inhibitor, effectively suppresses inflammatory cascades downstream of the IL12/IL23 axis in patients with IBD, psoriasis, and psoriatic arthritis. The high cost and side effects of biological therapy make it important to find a better treatment for these conditions in the future. Diet has a significant impact on the progression of CD, especially in first-world countries where a high rate of fast food that contains lots of inflammatory compounds is consumed. As a result, recent research has argued against using or eliminating certain dietary components for CD treatment [3].

In pediatric CD, exclusive enteral nutrition (EEN) is the sole recognized nutritional therapy, notably in Europe. In nearly 80% of patients, EEN causes clinical remission and supports gut repair [3]. The EEN mechanism is still unknown; however, research has revealed that gut flora modification drives its therapeutic properties [4]. Some other patients resumed their usual diet, but these benefits were reversed, and intestinal inflammation rose accordingly [5]. The goal of this systematic review is to investigate a treatment based on the diet of IBD patients. As a result, we may consider it a preventative therapeutic to minimize the recurrences of UC, CD, or its more common complications, such as Clostridium difficile infection.

## Review

Methodology

We did a systematic evaluation using free full-length papers and the Preferred Reporting Items checklist to describe our approach and results. This review was started on May 15, 2023.

Search Strategy

PubMed, Google Scholar, and Cochrane were used to collect databases using studies performed in the last two years using the following: Inflammatory bowel disease OR bloody diarrhea OR colitis (("Inflammatory Bowel Diseases/diet therapy"[Majr] OR "Inflammatory Bowel Diseases/prevention and control"[Majr])) OR ("Inflammatory Bowel Diseases/diet therapy"[Mesh:NoExp] OR "Inflammatory Bowel Diseases/prevention and control"[Mesh:NoExp]) AND Crohn's disease ("Crohn Disease/diet therapy"[Majr]) OR "Crohn Disease/diet therapy"[Majr:NoExp] AND Ulcerative colitis, bloody diarrhea ("Colitis, Ulcerative/diet therapy"[Majr]) OR "Colitis, Ulcerative/diet therapy" [Majr:NoExp].

Eligibility Criteria and Study Selection

To assess eligibility, two investigators carefully read the full title and content of each paper. We selected the latest literature and articles published in the past five years, including papers written in the English language or if a full free-text English-language translation is available. Articles were excluded if the full free text of the papers could not be retrieved. Articles focusing on the different diets used as optional therapeutics for IBD (gray literature and proposal papers) were also not included.

Quality Assessment

We used the Assessment of Multiple Systematic Reviews (AMSTAR) form and the Cochrane Risk of Bias assessment tools for clinical trials, systematic reviews, and meta-analyses.

Results

Search Results

**Figure 1:**
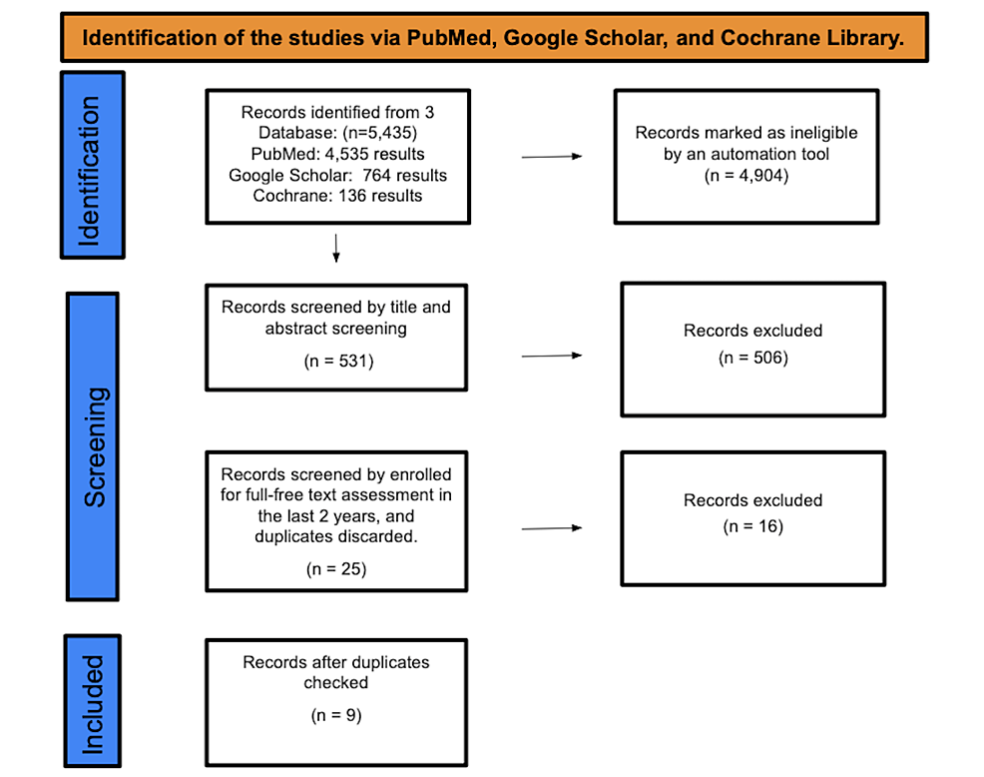
Identification of studies via databases and registers

A total of 5,435 studies were found after searching PubMed, Google Scholar, and Cochrane Library. A total of 4,904 were marked as ineligible by an automation tool. A total of 531 studies underwent title and abstract screening, and 506 papers were discarded. The remaining 25 papers were chosen for full-free text evaluation in the previous two years, and after discarding duplicates, resulting in the elimination of 16 studies, 9 studies were enlisted for the final collection of data (Figure 1).

Table 1 shows a summary and characteristics of all included studies.

**Table 1:** Table of data extraction RCT: randomised control trials; CD: *Clostridium difficile*; UC: ulcerative colitis; SRL: systematic review literature, FODMAP: Fermentable oligosaccharides, disaccharides, monosaccharides, and polyol.

Author	Year of publication	Study design	Quality tool	Primary research	Outcome evaluation
Keshteli et al. [6]	2022	RCT	Cochrane risk of bias assessment tool	RCT was conducted from 2014 to 2017 in patients between 18 and 75 years old.	The prevention of UC can be obtained by following an anti-inflammatory diet, mostly in patients with clinical remission.
Gonzalez et al. [7]	2022	SRL and Meta-analysis	AMSTAR checklist	5 relevant publications were obtained after methodology searching.	High clinical response using exclusive and partial enteral nutrition.
Lewis et al. [2]	2022	RCT	Cochrane risk of bias assessment tool	97 participants were selected and randomized for two different diets for 3,6,9, and 12 months.	Symptomatic remission was common with both diets.
Cox et al. [8]	2020	RCT	Cochrane risk of bias assessment tool	For one month, 51 participants were divided into groups and allocated to either a FODMAP diet or a normal diet.	FODMAP diet and regular follow-up are effective in the management of IBD.
Milajerdi et al. [9]	2020	RCT	Cochrane risk of bias assessment tool	30 patients in a low-FODMAP diet vs patients with an usual diet as control, for 1 month.	Low-FODMAP decreases IBD symptoms.
Comeche et al. [10]	2019	SRL and Meta-analysis	AMSTAR checklist	10 studies in total were included.	There is some evidence that fixed diets can help treat IBD, and they can be used with other medical treatments.
Svolos et al. [11]	2019	RCT	Cochrane risk of bias assessment tool	25 participants randomly received exclusive enteral nutrition or CD treatment for seven days.	Food-based diet is a treatment for active CD that can be used with enteral nutrition; particularly in adults.
Albenberg et al. [12]	2019	RCT	Cochrane risk of bias assessment tool	15,600 participants with inflammatory bowel disease completed a baseline survey.	The amount of red and processed meat eaten was not linked to how long it took for symptoms to come back.
Limketkai et al. [13]	2019	SRL	AMSTAR checklist	18 RCTs including 1878 participants	The dietary interventions on CD and UC were uncertain.

Discussion

We want to explain how dietary variables have been identified as possible environmental contributions to UC development in various experimental and epidemiological investigations. UC has been linked to a high diet of soft drinks and sugar, as well as a poor intake of fruits and vegetables [3,14]. 

In a study by Keshteli et al., six-month dietary RCT intervention research was conducted to investigate relapse prevention in UC, in which they followed a regular diet that may have a helpful effect in reducing the emergence of subclinical colitis. They also discovered substantial alterations in the serum, urine, and feces metabolomes. Compared to Canada's Food Guide dietary guidelines, significant alterations in particular fecal bacteria were detected after the antiinflammatory diet [6].

In the study by Gonzalez et al., it was found that there were no significant differences between EEN and PEN in terms of producing high response rates and clinical and analytical remission. PEN and EEN exhibited similar results, with excellent success rates and clinical remission with both regimens, indicating that PEN is at least as effective as EEN in generating remission of active Crohn's disease [7].

A RCT done by Lewis et al. examined the efficacy of specific carbohydrate diet (SCD) and Mediterranean diet (MD) in treating CD symptoms. While both diets decreased symptoms, we found no indication of an SCD advantage over MD in achieving symptomatic remission at six or twelve weeks, as well as no evidence of treatment effect heterogeneity depending on the presence or absence of verified inflammation during screening [2].

Cox et al. conducted a randomized, placebo-controlled experiment to show that low-FODMAP dietary guidance improves gastrointestinal symptoms and quality of life in patients with quiescent IBD when compared to sham dietary counseling. In an untargeted study, a low FODMAP diet did not affect the intestinal microbiota, but it did affect the immune components of the GI microbiota. Despite this, neither clinical disease activity nor inflammatory markers changed [8].

Milajerdi et al. showed that a low-FODMAP diet may decrease inflammation by modifying bacterial growth and will most likely limit IBD incidence and progression; however, this theory has yet to be validated in clinical studies. As a result, the lack of a clear therapy for IBD, as well as many drug-related problems, has raised attention to the use of alternative therapies [9].

Comeche et al. conducted an SRL meta-analysis. The fundamental idea of these diets was to reduce some pro-inflammatory foods while increasing others that are thought to maintain a healthy gut microbiota [10]. In light of the significant incidence of malnutrition, diets that may change the intestinal barrier and host immunity must be prioritized [2]. The aforementioned study did not find an increase in albumin, C-reactive protein, or fecal calprotectin levels, but they got good outcomes in Crohn's disease activity in patients with *Clostridium difficile* infection who were given preset diets, namely a microparticle diet, a semi-vegetarian diet, and an immunoglobulin exclusion diet [2]. It is good to let people know that, as a result, IBD-induced dysbiosis is characterized by decreased bacterial diversity, with the expansion of putative aggressive groups (such as Proteobacteria, Fusobacterium species, and *Ruminococcus gnavus*) combined with decreases in protective groups (such as Lachnospiraceae, Bifidobacterium species, Roseburia, and Sutterella), for which different diets will help in the formation of different bacteria colonies [15-20].

## Conclusions

Finally, we demonstrated that following a diet that reduces inflammation may prevent its recurrence in UC patients in clinical remission. Following this conclusion, there were significant systemic changes in the intestinal microbiota of anti-inflammatory diet patients. These results are promising, and they suggest that bigger, more rigorous randomized controlled trials of nutritional therapies for maintaining remission in UC patients are warranted.

CD patients with mild-to-severe symptoms tolerated MD and SCD well. Good symptomatic outcomes were prevalent with both diets, and they did not seem to be affected by the presence or lack of verified inflammation before randomization. We advise as well that a four-week FODMAP diet combined with professional counseling and regular follow-up will be helpful in the therapy of persistent gastrointestinal symptoms in quiescent IBD, although care should be used in the long run.
